# Prognostic factors and survival outcomes according to tumor subtype in patients with breast cancer lung metastases

**DOI:** 10.7717/peerj.8298

**Published:** 2019-12-17

**Authors:** Siying Chen, Jin Yang, Yang Liu, Haisheng You, Yalin Dong, Jun Lyu

**Affiliations:** 1 Department of Pharmacy, The First Affiliated Hospital of Xi’an Jiaotong University, Xi’an, China; 2 Clinical Research Center, The First Affiliated Hospital of Xi’an Jiaotong University, Xi’an, China; 3 School of Public Health, Xi’an Jiaotong University Health Science Center, Xi’an, Shaanxi, China

**Keywords:** Breast cancer, Lung metastases, Molecular subtype, Prognosis

## Abstract

**Background:**

Reports on the incidence and prognoses of lung metastases when diagnosing breast cancer patients with different subtypes are limited. Our study investigated the effect of molecular sub-typing stratification on the prognoses of lung metastatic breast caner patients.

**Methods:**

Patients with breast cancer and lung metastases were identified from Surveillance, Epidemiology and End Results population-based data between 2010 and 2015. Univariate and multivariate Cox regression analyses were performed to identify risk factors and prognoses, overall survival (OS) and breast cancer-specific survival for patients with breast cancer lung metastases.

**Results:**

We identified 6,516 patients with lung metastatic breast cancer, representing 1.7% of the entire cohort and 30.4% of the subset with metastatic disease. This included 2,940 hormone receptor (HR)+/HER2− patients, 852 HR+/HER2+ patients, 547 HR−/HER2+ patients and 983 triple-negative patients. The median OS for all lung metastatic patients was 13 months. Multivariate analysis revealed that those lung metastatic breast cancer patients of older age (>80), black race, with poorly differentiated tumors, carcinoma histology, triple-negative subtype, more metastatic sites and no surgery, and no chemotherapy showed significantly poor survival, both overall and breast cancer-specific.

**Conclusions:**

Our findings show that molecular sub-type and more metastatic sites might have significant influence on the incidence and prognosis of breast cancer lung metastases. We also identified several prognostic factors that could guide therapy selection in the treatment of lung metastatic patients.

## Introduction

Breast carcinoma is the most common cancer diagnosed among women worldwide, with more than one in 10 new breast cancer cases occurring each year. After lung cancer, it is the second leading cause of cancer-related mortality among females (DeSantis et al., 2017; Ferlay et al., 2015). Moreover, metastatic disease, or the spread of tumor cells throughout the body, is responsible for the great majority of breast cancer patient deaths (Redig & McAllister, 2013; Scully et al., 2012; Yousefi et al., 2018). It has been reported that about 20–30% of patients develop metastatic disease when diagnosed with early breast cancer (Early Breast Cancer Trialists’ Collaborative Group, 2005; Soni et al., 2015). Breast cancer has a propensity for specific organs, most frequently bone and lung and in a lower extents, the liver and brain (Landemaine et al., 2008; Minn et al., 2005; Soni et al., 2015). A previous study showed that approximately 60% of breast cancer patients suffered lung or bone metastasis in their life (Gennari et al., 2005).

Breast cancer lung metastatic patients have a median survival rate of only 22 months after treatment, indicating poor prognoses (Smid et al., 2008). It has been reported that 60–70% of breast cancer patients who eventually died were diagnosed with lung metastasis (Jin et al., 2018). Although a variety of available treatments for lung metastasis are being improved, such as radiotherapy, chemotherapy, or targeted therapy, the survival rate for lung metastatic patients remains very low. This seriously endangers the patient’s quality of life and even life expectancy.

The main subtypes of breast cancer are divided by the expression statuses of three tumor markers evaluated comprehensively and routinely because of their application in guiding clinical treatment: estrogen receptor, progesterone receptor and human epidermal growth factor 2-neu (HER2). Among all breast cancers, approximately 75% of cases are classified as hormone receptor-positive (HR+; ER+ and/or the PR+) and HER2-negative (Howlader et al., 2014) and the statuses of ER and PR are predicted by the response to endocrine therapy (Ali, Buluwela & Coombes, 2011). HER2-positive cases represent 20% of breast cancers and show more aggressive clinical outcome because of restrictions on the application of chemotherapy (Awada et al., 2016). Additionally, triple negative breast cancers (TNBC), where there is an absence of ER, PR, and HRE2, account for 10–15% of all breast cancer cases. Compared with other breast cancer subtypes, TNBC is characterized by early relapse and aggressive behavior, with HER2-positive metastatic breast cancer specifically considered an incurable disease with the worst prognosis (Omarini et al., 2018).

Metastatic breast cancer patients with various molecular subtypes have different clinical characteristics and prognoses, and the treatments they receive are also different. Patients with lung metastases account for a great proportion of metastatic breast cancer cases, and the influence of different tumor subtypes on the survival of lung metastatic patients is not clear. Therefore, it is necessary to analyze prognostic factors and survival outcomes for lung metastatic breast cancer patients in order to provide some basis for clinical treatment.

The purpose of this study was to use surveillance, epidemiology, and end results (SEER) population-based data to characterize the incidence of lung metastases at the time of initial diagnosis among stage IV breast cancer patients. We also assessed related risk factors and survival outcomes to improve the prognosis of lung metastatic breast cancer and reduce the occurrence of lung metastasis.

## Materials and Methods

### Data source and study design

The population-based data were obtained from SEER, and included cancer incidence and survival data from 18 registries. Within the SEER database, we extracted 379,261 patients who were diagnosed with breast cancer between 1st January 2010 and 31st December 2015. We then generated a cohort of 21,435 stage IV breast cancer patients. Of these, 6,516 patients were diagnosed with lung metastases. We excluded patients who were diagnosed at autopsy or by death certificate, as well as patients whose follow-up times were unknown. Finally, we identified 5,760 patients eligible for survival analysis. This study was exempted by the Ethics Committee of the First Affiliated Hospital of Xi’an Jiaotong University, because all data extracted from the publicly available SEER database were free and recognized as nonhuman studies (Cao et al., 2019; Ye et al., 2019).

Patients were classified by breast cancer molecular subtypes: HR-positive/HER2-negative (HR+/HER2−), HR-positive/HER2-positive (HR+/HER2+), HR-negative/HER2-positive (HR−/HER2+) and triple-negative (HR-negative/HER2-negative). Study variables included age at diagnosis, gender, race, marital status, tumor sites, tumor grade, histology, number of other metastatic sites, surgery, radiation therapy, chemotherapy, and vital status. Other sites of metastasis were defined as different metastasis at initial diagnosis of bone, liver, or brain.

### Statistical analysis

Clinical characteristics of the different subtypes were compared with chi-square or Fisher’s exact tests. We used the Kaplan–Meier method to estimate survival probabilities and to generate survival curves within various subtypes and the number of other metastatic sites. A Cox proportional hazards regression was conducted to identify the independent association of variables with overall survival (OS) and BCSS in lung metastatic patients. Hazard ratios and 95% confidence intervals (95% CIs) were calculated using the Cox model. All *P* values of 0.05 or less were considered statistically significant, and *P* values were two-tailed. Statistical analyses were performed using SPSS statistical software (version 24.0; IBM Corporation, Armonk, NY, USA).

## Results

### Incidence

A total of 379,261 patients diagnosed with breast cancer were classified by different subtypes to analyze incidence and median survival between 2010 and 2015 (Table 1). The incidence proportions of HR+/HER2−, HR+/HER2+, HR−/HER2+, triple-negative and unknown subtype were 66.9%, 9.6%, 4.1%, 10.3% and 9.0%, respectively. Among the 21,435 patients with metastatic disease at any site (bone, lung, liver, brain), 50.9%, 13.4%, 7.3%, 11.5% and 16.9% had HR+/HER2−, HR+/HER2+, HR−/HER2+, triple-negative and unknown subtype, respectively. In the entire cohort, the 6,516 patients diagnosed with lung metastases accounted for 1.7% of the entire cohort, and 30.4% of the subset with metastatic disease. Of these, the incidence proportions were higher among patients with HR−/HER2+ molecular subtype (3.5% of the entire cohort and 35.1% of the metastatic subgroup) and triple-negative subtype (2.5% of the entire cohort and 39.7% of the metastatic subgroup).

**Table 1:** The incidence proportion and median survival of breast cancer patients with lung metastases stratified by subtypes.

Subtypes	Patients No.			Incidence proportion of lung metastases, %		Survival among patients with lung metastases, median (IQR), months
	With breast cancer	With metastatic disease	With lung metastases	Among entire cohort	Among subset with metastatic disease	
HR+/HER2−	253,783	10,909	2,940	1.2	27.0	16.0 (6.0–31.0)
HR+/HER2+	36,458	2,872	852	2.3	29.7	17.0 (7.0–31.0)
HR−/HER2+	15,727	1,557	547	3.5	35.1	12.0 (5.0–25.0)
Triple-negative	39,147	2,473	983	2.5	39.7	8.0 (3.0–16.0)
Unknown	34,146	3,624	1,194	3.5	32.9	8.0 (2.0–22.0)
All subtypes	379,261	21,435	6,516	1.7	30.4	13.0 (5.0–27.0)

We assessed the distribution of clinical characteristics of lung metastatic patients according to their molecular subtypes. There were significant differences among the entire cohort (Table 2). The lung metastatic patients from the HR+/HER2− subtype showed older age at diagnosis (*P* < 0.001), lower-grade tumors (*P* < 0.001), high proportions of lobular histology (*P* < 0.001) and two metastatic sites at initial diagnosis (*P* < 0.001). Patients with the HR+/HER2+ subtype had high proportions of ductal histology (*P* < 0.001), three metastatic sites at initial diagnosis (*P* < 0.001) and more survivors (*P* < 0.001). The HR−/HER2+ cases were aged younger at diagnosis (*P* < 0.001), had four metastatic sites at initial diagnosis (*P* < 0.001) and high proportions of undergoing chemotherapy (*P* < 0.001). Patients with triple-negative subtype, by contrast, had higher-grade tumors (*P* < 0.001) and higher proportions of receiving surgery and radiation (*P* < 0.001). The triple-negative patients also had higher rates of lung metastases and poorer survival rates (*P* < 0.001).

**Table 2:** The clinical characteristics of patients with lung metastases according to tumor subtypes.

Patients characteristics	Tumor subtypes						*P* value
	HR+/HER2−	HR+/HER2+	HR−/HER2+	Triple-negative	Unknown	Total	
	*n* (%)	*n* (%)	*n* (%)	*n* (%)	*n* (%)	*n* (%)	
All patients	2,940 (45.1)	852 (13.1)	547 (8.4)	983 (15.1)	1,194 (18.3)	6,516 (100)	
**Age at diagnosis, years**							<0.001
18–40	137 (4.7)	62 (7.3)	51 (9.3)	75 (7.6)	33 (2.8)	358 (5.5)	
41–60	977 (33.2)	370 (43.4)	255 (46.6)	369 (37.5)	326 (27.3)	2,297 (35.3)	
61–80	1,465 (49.8)	340 (39.9)	197 (36.0)	419 (42.6)	538 (45.1)	2,959 (45.4)	
>80	361 (12.3)	80 (9.4)	44 (8.0)	120 (12.2)	297 (24.9)	902 (13.8)	
**Gender**							0.052
Male	54 (1.8)	16 (1.9)	2 (0.4)	8 (0.8)	18 (1.5)	98 (1.5)	
Female	2,886 (98.2)	836 (98.1)	545 (99.6)	975 (99.2)	1,176 (98.5)	6,418 (98.5)	
Race							<0.001
White	2,168 (73.7)	627 (73.6)	378 (69.1)	646 (65.7)	910 (76.2)	4,729 (72.6)	
Black	508 (17.3)	151 (17.7)	107 (19.6)	260 (26.4)	207 (17.3)	1,233 (18.9)	
Other	253 (8.6)	72 (8.5)	60 (11.0)	76 (7.7)	70 (5.9)	531 (8.1)	
Unknown	11 (0.4)	2 (0.2)	2 (0.4)	1 (0.1)	7 (0.6)	23 (0.4)	
**Marital status**							0.033
Single	678 (23.1)	193 (22.7)	116 (21.2)	209 (21.3)	289 (24.2)	1,485 (22.8)	
Married/domestic partner	1,132 (38.5)	363 (42.6)	223 (40.8)	390 (39.7)	396 (33.2)	2,504 (38.4)	
Divorced/separated/widowed	958 (32.6)	251 (29.5)	170 (31.1)	329 (33.5)	433 (36.3)	2,141 (32.9)	
Unknown	172 (5.9)	45 (5.3)	38 (6.9)	55 (5.6)	76 (6.4)	386 (5.9)	
**Tumor site(s)**							<0.001
Only one site (left or right)	2,853 (97.0)	837 (98.2)	531 (97.1)	958 (97.5)	1,050 (87.9)	6,229 (95.6)	
Two or more sites	87 (3.0)	15 (1.8)	16 (2.9)	25 (2.5)	144 (12.1)	287 (4.4)	
**Tumor grade**							<0.001
I	228 (7.8)	20 (2.3)	1 (0.2)	9 (0.9)	23 (1.9)	281 (4.3)	
II	1,191 (40.5)	274 (32.2)	90 (16.5)	110 (11.2)	166 (13.9)	1,831 (28.1)	
III	937 (31.9)	424 (49.8)	347 (63.4)	702 (71.4)	197 (16.5)	2,607 (40.0)	
IV	16 (0.5)	7 (0.8)	9 (1.6)	14 (1.4)	11 (0.9)	57 (0.9)	
Unknown	568 (19.3)	127 (14.9)	100 (18.3)	148 (15.1)	797 (66.8)	1,740 (26.7)	
**Histology**							<0.001
Ductal	2,176 (74.0)	700 (82.2)	434 (79.3)	726 (73.9)	418 (35.0)	4,455 (68.4)	
Lobular	176 (6.0)	13 (1.5)	2 (0.4)	11 (1.1)	37 (3.1)	239 (3.7)	
Mixed ductal and lobular	165 (5.6)	37 (4.3)	11 (2.0)	20 (2.0)	19 (1.6)	252 (3.9)	
Mucinous	52 (1.8)	5 (0.6)	0 (0)	1 (0.1)	5 (0.4)	63 (1.0)	
Carcinoma	371 (12.6)	97 (11.4)	100 (18.3)	224 (22.8)	715 (59.9)	1,507 (23.1)	
**Extrapulmonary metastatic sites to bone, brain, and liver, No.**							<0.001
0	831 (28.3)	259 (30.4)	212 (38.8)	448 (45.6)	394 (33.0)	2,144 (32.9)	
1	1,420 (48.3)	314 (36.9)	159 (29.1)	339 (34.5)	470 (39.4)	2,702 (41.5)	
2	587 (20.0)	241 (28.3)	136 (24.9)	144 (14.6)	257 (21.5)	1,365 (20.9)	
All 3	87 (3.0)	36 (4.2)	37 (6.8)	43 (4.4)	36 (3.0)	239 (3.7)	
Unknown	15 (0.5)	2 (0.2)	3 (0.5)	9 (0.9)	37 (3.1)	66 (1.0)	
**Cancer-directed surgery**							<0.001
Performed	583 (19.8)	212 (24.9)	168 (30.7)	332 (33.8)	123 (10.3)	1,418 (21.8)	
Not performed	2,307 (78.5)	627 (73.6)	366 (66.9)	635 (64.6)	1,043 (87.4)	4,978 (76.4)	
Unknown	50 (1.7)	13 (1.5)	13 (2.4)	16 (1.6)	28 (2.3)	120 (1.8)	
**Radiation**							<0.001
Yes	790 (26.9)	220 (25.8)	150 (27.4)	279 (28.4)	156 (13.1)	1,595 (24.5)	
No	2,107 (71.7)	616 (72.3)	385 (70.4)	687 (69.9)	1,034 (86.6)	4,829 (74.1)	
Unknown	42 (1.4)	16 (1.9)	12 (2.2)	17 (1.7)	4 (0.3)	91 (1.4)	
**Chemotherapy**							<0.001
Yes	1,241 (42.2)	610 (71.6)	410 (75.0)	683 (69.5)	304 (25.5)	3,248 (49.8)	
No/unknown	1,699 (57.8)	242 (28.4)	137 (25.0)	300 (30.5)	890 (74.5)	3,268 (50.2)	
**Status**							<0.001
Alive	1,335 (45.4)	460 (54.0)	245 (44.8)	230 (23.4)	254 (21.3)	2,524 (38.7)	
Dead	1,605 (54.6)	392 (46.0)	302 (55.2)	753 (76.6)	940 (78.7)	3,992 (61.3)	
**Cause of death**							<0.001
Alive	1,335 (45.4)	460 (54.0)	245 (44.8)	230 (23.4)	254 (21.3)	2,524 (38.7)	
Breast cancer	1,357 (46.2)	343 (40.3)	261 (47.7)	669 (68.1)	740 (62.0)	3,370 (51.7)	
Other	248 (8.4)	49 (5.8)	41 (7.5)	84 (8.5)	200 (16.8)	622 (9.5)	

### Survival

**Figure 1:**
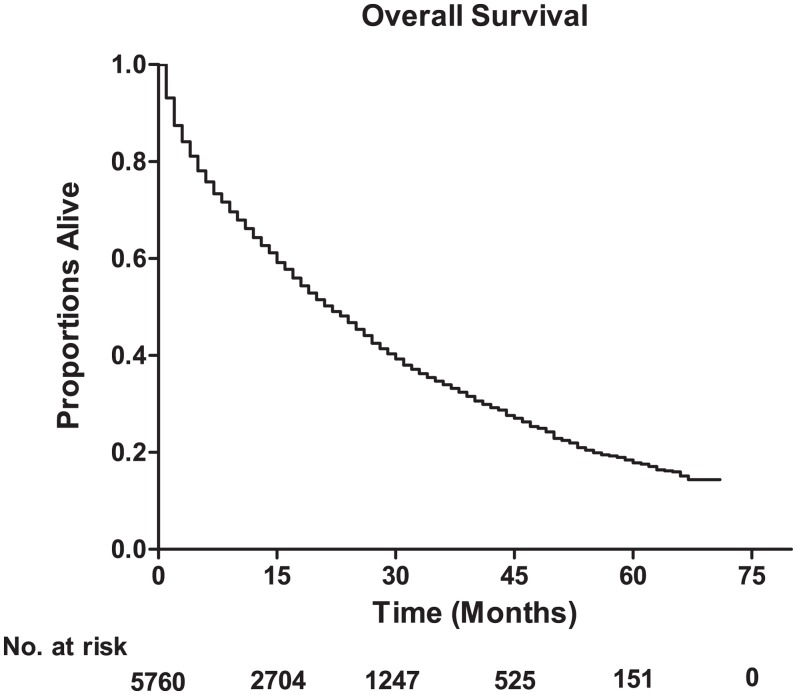
The overall survival for the patients with lung metastases from breast cancer, and the table showing the number at risk for overall survival.

**Figure 2:**
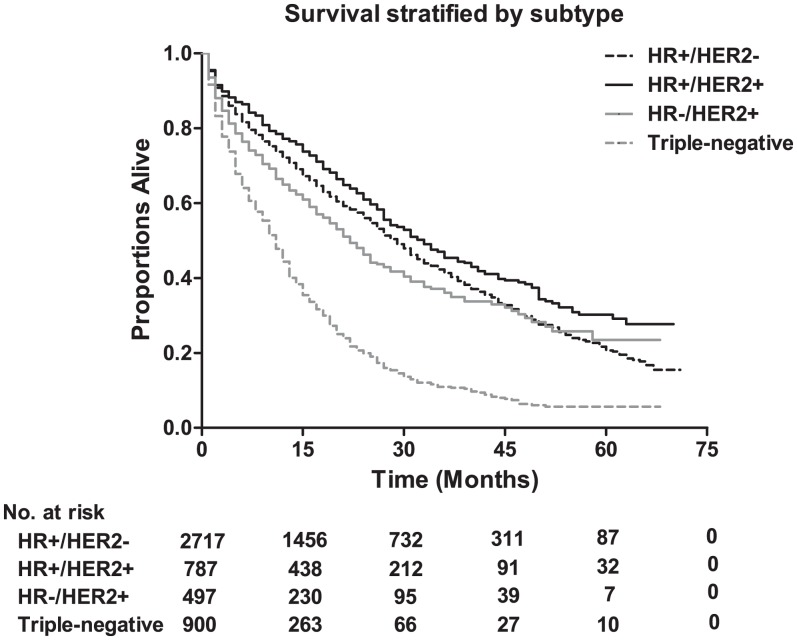
The overall survival according to tumor subtype, and the table showing the number at risk for tumor subtype. Log-rank *P* < 0.0001. HR, hormone receptor; HER2, human epidermal growth factor receptor 2.

**Figure 3:**
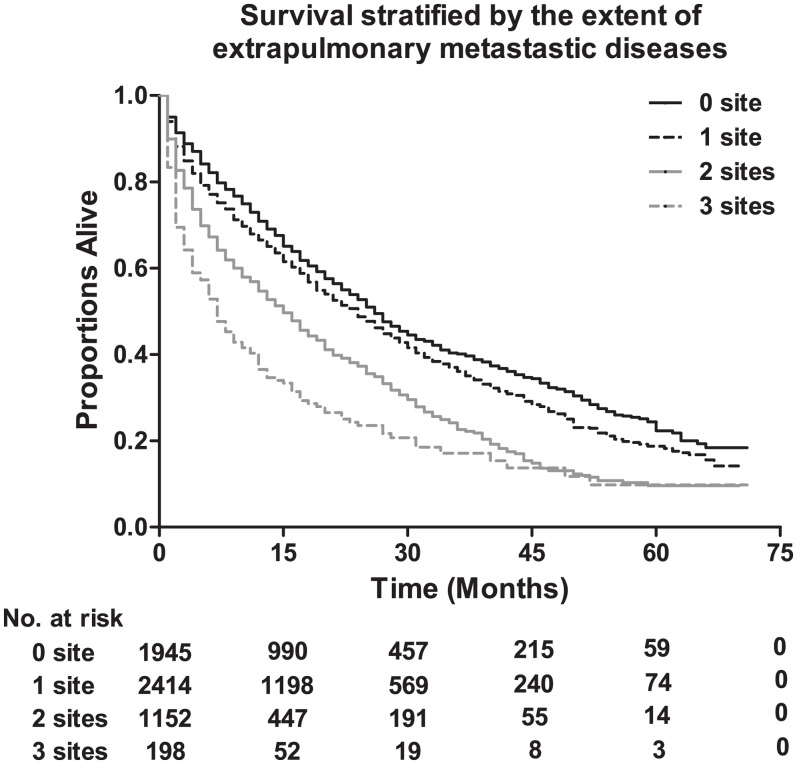
The overall survival stratified by the extent of extrapulmonary metastatic disease, and the table showing the number at risk according to number of metastasis sites. Log-rank *P* < 0.0001.

The median survival among lung metastatic patients was 13 months, and the 3-year and 5-year survival rates of these patients were 33.9% and 17.8%, respectively (Fig. 1). Among the four subtypes shown in Fig. 2, patients with the HR+/HER2+ subtype had the longest median survival (17 months), and their 3-year and 5-year survival rates were 45.4% and 30.2%, respectively. Triple-negative subtypes had the shortest median survival (8 months), and their 3-year and 5-year survival rates were 10.9% and 5.6%, respectively. Moreover, an exploratory analysis of OS according to the number of metastatic tumor sites showed significant differences among lung metastatic patients. The cases with only lung metastases had longer median survival (15 months) compared with patients with four metastasis sites at initial diagnosis (6 months). The 3-year survival rate of patients with only lung metastases and four metastasis sites were 40.1% and 17.1%, and the 5-year survival rate of these were 22.3% and 9.8%, respectively (Fig. 3).

We used a Cox proportional hazards model to investigate prognostic factors for OS and BCSS using univariate and multivariate analysis. In the univariate model for OS, patients with poor prognoses were older (age >80; HR, 2.418; (95% CI [2.022–2.893]); *P* < 0.001), black (HR, 1.253; (95% CI [1.152–1.363]); *P* < 0.001), had tumor grade III (HR, 1.890; (95% CI [1.557–2.294]); *P* < 0.001), were triple-negative subtype (HR, 2.353; (95% CI [2.145–2.581]); *P* < 0.001) and had four metastasis sites at initial diagnosis (HR, 2.345; (95% CI [1.972–2.790]); *P* < 0.001). BCSS results were consistent with those for OS (Table 3). Furthermore, using multivariate analysis, we found that the dependent prognostic factors for OS were older age, black race, tumor grade III, triple-negative subtype, having four metastasis sites at initial diagnosis, non-surgery, and not receiving chemotherapy. The results for BCSS exhibited a similar trend (Table 4).

**Table 3:** Unadjusted overall survival and breast cancer-specific survival.

Variable	Survival, median (IQR), months	Overall survival			Breast cancer-specific survival		
		Log-rank *P*	Hazard ratio [95% CI]	*P* value	Log-rank *P*	Hazard ratio [95% CI]	*P* value
**Age at diagnosis, years**							
18–40	18.0 (8.0–30.0)	<0.001	Reference		<0.001	Reference	
41–60	15.0 (6.0–29.0)		1.236 [1.045–1.461]	0.013		1.243 [1.040–1.485]	0.017
61–80	13.0 (4.0–27.0)		1.454 [1.233–1.715]	<0.001		1.379 [1.157–1.644]	<0.001
>80	7.0 (2.0–18.0)		2.418 [2.022–2.893]	<0.001		2.111 [1.741–2.561]	<0.001
**Sex**							
Male	17.0 (4.0–32.0)	0.059	Reference		0.016	Reference	
Female	13.0 (5.0–27.0)		1.324 [0.983–1.783]	0.065		1.508 [1.070–2.126]	0.019
**Race**							
White	13.0 (5.0–28.0)	<0.001	Reference		<0.001	Reference	
Black	12.0 (4.0–24.0)		1.253 [1.152–1.363]	<0.001		1.262 [1.153–1.383]	<0.001
Other	14.0 (5.0–28.0)		0.880 [0.770–1.004]	0.058		0.851 [0.736–0.985]	0.030
**Marital status**							
Single	13.0 (4.0–27.0)	<0.001	Reference		<0.001	Reference	
Married/domestic partner	15.0 (6.0–30.0)		0.768 [0.702–0.840]	<0.001		0.781 [0.709–0.860]	<0.001
Divorced/separated/widowed	10.0 (4.0–23.0)		1.134 [1.036–1.240]	0.006		1.097 [0.995–1.209]	0.063
**Tumor site(s)**							
Only one site (left or right)	13.0 (5.0–27.0)	<0.001	Reference		0.073	Reference	
Two or more sites	8.0 (2.0–22.0)		1.346 [1.138–1.591]	0.001		1.189 [0.981–1.441]	0.078
**Tumor grade**							
I	18.0 (6.0–32.0)	<0.001	Reference		<0.001	Reference	
II	17.0 (6.0–31.0)		1.247 [1.023–1.521]	0.029		1.310 [1.049–1.634]	0.017
III	12.0 (5.0–25.0)		1.890 [1.557–2.294]	<0.001		2.100 [1.692–2.606]	<0.001
IV	14.0 (4.0–28.0)		1.785 [1.204–2.646]	0.004		1.954 [1.277–2.990]	0.002
**Histology**							
Ductal	14.0 (5.0–28.0)	<0.001	Reference		<0.001	Reference	
Lobular	18.0 (6.0–30.0)		1.048 [0.879–1.249]	0.603		1.020 [0.843–1.234]	0.603
Mixed ductal and lobular	18.0 (7.0–33.0)		0.782 [0.652–0.939]	0.009		0.764 [0.627–0.931]	0.008
Mucinous	16.0 (5.0–34.0)		0.658 [0.440–0.983]	0.041		0.629 [0.405–0.978]	0.629
Carcinoma	8.0 (3.0–21.0)		1.563 [1.441–1.694]	<0.001		1.438 [1.315–1.573]	<0.001
**Tumor subtype**							
HR+/HER2−	16.0 (6.0–31.0)	<0.001	Reference		<0.001	Reference	
HR+/HER2+	17.0 (7.0–31.0)		0.821 [0.730–0.923]	0.001		0.847 [0.747–0.960]	0.009
HR−/HER2+	12.0 (5.0–25.0)		1.171 [1.026–1.337]	0.019		1.184 [1.026–1.336]	0.021
Triple-negative	8.0 (3.0–16.0)		2.353 [2.145–2.581]	<0.001		2.478 [2.245–2.736]	<0.001
**Extrapulmonary metastatic sites to bone, brain, and liver, No.**							
0	15.0 (6.0–28.0)	<0.001	Reference		<0.001	Reference	
1	14.0 (5.0–28.0)		1.153 [1.063–1.251]	0.001		1.199 [1.097–1.310]	<0.001
2	10.0 (3.0–23.0)		1.654 [1.506–1.818]	<0.001		1.771 [1.599–1.960]	<0.001
All 3	6.0 (2.0–16.0)		2.345 [1.972–2.790]	<0.001		2.411 [1.996–2.913]	<0.001
**Cancer-directed surgery**							
Performed	19.0 (8.0–34.0)	<0.001	Reference		<0.001	Reference	
Not performed	11.0 (4.0–25.0)		1.613 [1.484–1.754]	<0.001		1.557 [1.424–1.703]	<0.001
**Radiation**							
Yes	14.0 (5.0–28.0)	0.640	Reference		0.716	Reference	
No	13.0 (4.0–27.0)		1.018 [0.943–1.099]	0.647		0.985 [0.908–1.069]	0.721
**Chemotherapy**							
Yes	15.0 (6.0–28.0)	<0.001	Reference		<0.001	Reference	
No/unknown	11.0 (3.0–25.0)		1.424 [1.331–1.524]	<0.001		1.321 [1.227–1.422]	<0.001

**Table 4:** Multivariate analysis overall survival and breast cancer-specific survival.

Variable	Overall survival		Breast cancer-specific survival	
	Hazard ratio [95% CI]	*P* value	Hazard ratio [95% CI]	*P* value
**Age at diagnosis, years**				
18–40	Reference		Reference	
41–60	1.236 [1.012–1.509]	0.038	1.277 [1.034–1.577]	0.023
61–80	1.491 [1.217–1.825]	<0.001	1.479 [1.193–1.832]	<0.001
>80	2.189 [1.720–2.784]	<0.001	2.107 [1.628–2.727]	<0.001
**Sex**				
Male	Reference		Reference	
Female	1.177 [0.808–1.715]	0.397	1.306 [0.855–1.996]	0.217
**Race**				
White	Reference		Reference	
Black	1.246 [1.112–1.397]	<0.001	1.221 [1.081–1.379]	0.001
Other	0.982 [0.836–1.154]	0.829	0.943 [0.792–1.123]	0.509
**Marital status**				
Single	Reference		Reference	
Married/domestic partner	0.805 [0.717–0.905]	<0.001	0.794 [0.702–0.898]	<0.001
Divorced/separated/widowed	1.048 [0.928–1.185]	0.449	1.020 [0.895–1.162]	0.764
**Tumor site(s)**				
Only one site (left or right)	Reference		Reference	
Two or more sites	1.584 [0.976–2.572]	0.063	1.733 [1.051–2.857]	0.031
**Tumor grade**				
I	Reference		Reference	
II	1.301 [1.044–1.620]	0.019	1.374 [1.075–1.756]	0.011
III	1.865 [1.492–2.330]	<0.001	2.070 [1.615–2.652]	<0.001
IV	1.635 [1.037–2.580]	0.034	1.796 [1.099–2.934]	0.019
**Histology**				
Ductal	Reference		Reference	
Lobular	0.913 [0.714–1.169]	0.471	0.922 [0.708–1.202]	0.55
Mixed ductal and lobular	1.056 [0.860–1.298]	0.603	1.058 [0.847–1.322]	0.617
Mucinous	0.765 [0.439–1.332]	0.343	0.763 [0.418–1.394]	0.379
Carcinoma	1.412 [1.218–1.636]	<0.001	1.364 [1.165–1.598]	<0.001
**Tumor subtype**				
HR+/HER2−	Reference		Reference	
HR+/HER2+	0.906 [0.786–1.045]	0.175	0.914 [0.786–1.063]	0.244
HR−/HER2+	1.272 [1.073–1.508]	0.005	1.232 [1.027–1.478]	0.025
Triple-negative	2.766 [2.436–3.140]	<0.001	2.872 [2.510–3.285]	<0.001
**Extrapulmonary metastatic sites to bone, brain, and liver, No.**				
0	Reference		Reference	
1	1.477 [1.323–1.649]	<0.001	1.512 [1.344–1.702]	<0.001
2	2.429 [2.137–2.760]	<0.001	2.489 [2.171–2.853]	<0.001
All 3	3.833 [3.022–4.862]	<0.001	3.950 [3.072–5.080]	<0.001
**Cancer-directed surgery**				
Performed	Reference		Reference	
Not performed	1.541 [1.389–1.710]	<0.001	1.519 [1.360–1.697]	<0.001
**Radiation**				
Yes	Reference		Reference	
No	1.047 [0.949–1.154]	0.362	1.047 [0.943–1.162]	0.393
**Chemotherapy**				
Yes	Reference		Reference	
No/unknown	1.457 [1.313–1.618]	<0.001	1.420 [1.270–1.588]	<0.001

## Discussion

Lung metastasis is the most frequent type of breast cancer metastasis after bone metastasis. Few symptoms usually emerge until after the lungs have been replaced by metastatic tumor cells (Rashid & Takabe, 2012; Yousefi et al., 2018). Because it is difficult to discover from the surface, lung metastasis has been a huge challenge in the treatment of breast cancer patients. On account of the particularity of breast cancer, treatments are different based on the various molecular subtypes (Smid et al., 2008). Current treatment strategies, including surgical resection, chemotherapy and/or radiotherapy, provide relief rather than cure, especially for patients diagnosed with Stage IV disease (Redig & McAllister, 2013). In this study, lung metastatic patients diagnosed initially with breast cancer were classified by different tumor subtypes, and their incidence and dependent prognostic factors were explored. We identified 6,516 patients with lung metastases with initially diagnosed breast cancer, accounting for 1.7% of the entire cohort and 30.4% of metastatic disease subgroups, respectively. Similarly, Savci-Heijink et al. (2015) reported that lung metastases accounted for 31.4% of all metastases in breast cancer. Our results suggested that the incidence proportion of lung metastases was higher among patients with triple-negative and HR−/HER2+ subtypes. The incidence of lung metastasis was also shown to reach up to 40% in triple-negative breast cancer compared with only 20% in non-triple-negative breast cancer, and our results were similar to previous studies (Jin et al., 2018; Kim, Jung & Koo, 2014; Soni et al., 2015).

In the present study, median survival from breast cancer diagnoses obviously varied by subtype, ranging from 8 months in triple-negative patients to 17 months in HR+/HER2+ patients. The median survival of all lung metastatic patients was 13 months, which was shorter than the 22 or 32 months reported in lung metastatic patients after treatment or metastasectomy in previous studies (Jin et al., 2018; Macherey et al., 2017; Welter et al., 2008). Although patients with lung metastases had poor prognoses, it was noteworthy that the 3-year and 5-year survival rate of patients was up to 33.9% and 17.8%, respectively. Prior studies have shown that the 5-year survival rate of lung metastatic patients from the first pulmonary metastasectomy was 36% or 30.8% (Macherey et al., 2017; Welter et al., 2008). Few studies have been able to reveal the association between the survival rate of breast cancer patients with lung metastases and various molecular subtypes. However, our study identified that patients with HR+/HER2+ subtype showed the longest OS, and the 5-year survival rate was up to 30.2%. These findings were similar to a previous report on metastatic breast cancer (Lobbezoo et al., 2013). We found that the triple-negative subtype had the worst prognosis, and its 5-year survival rate was only 5.6%. Sundquist, Brudin & Tejler (2017) found that only 23% of patients with metastatic breast cancer of the triple-negative subtype survived 2 years or longer. The great differences in prognoses observed in all tumor subtypes demonstrates that breast cancer is a heterogeneous disease. In our study, we saw that patients with lung metastases only showed a lower risk of death than cases with four metastatic sites at initial diagnosis, indicating that there is a significant reduction in OS as the number of involved sites increases. Our findings were similar to previous reports on the impact of metastatic sites on the OS of breast cancer patients (Leone et al., 2017). Additionally, some comprehensive studies of four metastatic sites (bone, lung, liver and brain) suggested that patients with brain metastases had significantly poor survival when compared with patients with other metastases (Gong et al., 2017; Leone et al., 2017; Wang et al., 2017).

Our results revealed that older age, black race, advanced tumor grade, triple-negative subtype and number of metastasis sites were all independent risk factors. We also found the hormone receptor status was a vital prognostic factor that should not be ignored. Patients with HR−/HER2+ subtypes did not show a survival advantage when compared with HR+/HER2+ patients. This may be because endocrine therapy plays a significant role in ER-positive patients with breast cancer. Endocrine therapy could improve disease control, symptom relief, and quality of life in most hormone-receptor positive metastatic breast cancer patients (El Saghir et al., 2011). Additionally, chemotherapy combined with endocrine therapy could be significantly better than endocrine therapy alone for these patients (Early Breast Cancer Trialists’ Collaborative Group, 2005). Chemotherapy is generally used to periodically treat metastatic breast cancer patients with negative hormonal receptors because of its acceptable and reversible toxicity on normal cells (El Saghir et al., 2011). Triple-negative breast cancer does not respond to hormone therapy or other available targeted agents, and so chemotherapy became the mainstay in the treatment of patients with breast cancer (Hudis & Gianni, 2011). Because of the unique molecular profile, aggressive behavior of the cancer, and easy relapse in triple-negative patients, more clinical trials are developing and applying targeted drugs, such as epidermal growth factor receptor, vascular endothelial growth factor and poly (ADP-ribose) polymerase (PARP) inhibitors (Anders & Carey, 2008; Andre & Zielinski, 2012). We hope more possibilities for a metastatic breast cancer cure can developed in the near future.

Some limitations exist in our research. First, the SEER database does not include information on disease recurrence or subsequent lung metastases. Thus, we could only depict the presence or absence of lung metastases at initial diagnosis, and not comment on patients who developed lung metastases during disease progression. Second, our study was not able to investigate the impact of lung-directed treatment (such as pulmonary metastasectomy or endocrine therapy) on the prognoses of patients, because the public SEER database does not provide this information. Further studies using alternative data should be performed to address this significant point. Third, other metastasis sites such as lymph nodes, peritoneum, pleura or skin which may be helpful in analyzing the prognoses of metastatic breast cancer, were not collected in our study. Fourth, the retrospective nature of the study has an inherent bias.

## Conclusions

Our research provides crucial insight into the incidence, prognostic assessment, and risk stratification of breast cancer with lung metastases, enriching the study of the epidemiology of this occurrence. The tumor molecular subtypes and specific sites of extrapulmonary metastases could be used for prognostic evaluation in other prospective studies, and will provide guidance in the early diagnosis and effective treatment of lung metastases in breast cancer.

## Supplemental Information

10.7717/peerj.8298/supp-1 Supplemental Information 1 Raw data.

## Additional Information and Declarations

### Data Availability

The following information was supplied regarding data availability: The raw data is available as a Supplemental File.
